# An overview of new translational, clinical and therapeutic perspectives in laminopathies and other nuclear envelope-related diseases.

**DOI:** 10.1186/1750-1172-10-S2-I1

**Published:** 2015-11-11

**Authors:** Annachiara De Sandre-Giovannoli, Nicolas Levy, Rabah Ben Yaou, France Leturcq, Giovanna Lattanzi, Gisèle Bonne

**Affiliations:** 1 Aix Marseille Université, INSERM, GMGF UMR_S 910 & Department of Medical Genetics and Cell Biology, La Timone Children's hospital, APHM, 13385, Marseille, France; 2 Sorbonne Universités, UPMC Univ Paris 06, INSERM UMRS974, CNRS FRE3617, Center for Research in Myology, F-75013 Paris, France; 3 Institut de Myologie, F-75013, Paris, France; 4 AP-HP, Groupe Hospitalier Cochin-Broca-Hôtel Dieu, Laboratoire de biochimie et génétique moléculaire, Paris, France; 5 CNR Institute for Molecular Genetics, Unit of Bologna, Bologna, Italy; 6 Rizzoli Orthopedic Institute, Laboratory of Musculoskeletal Cell Biology, Bologna, Italy

## Introduction

Defects of proteins of the nuclear envelope are now recognized as a vast group of heterogeneous rare inherited diseases. The first reported nuclear envelope-related disease, has been the X-linked form of the Emery-Dreifuss muscular dystrophy (EDMD1, OMIM#310300), a condition characterized by muscle weakness and wasting usually with a humeroperoneal distribution in the first stages, early joint contractures of Achilles tendons, elbows, neck and spine, and cardiac involvement featuring conduction defects, arrhythmias, subsequent dilated cardiomyopathy and frequently responsible of sudden death. EDMD1 is due to mutation of the *EMD* gene encoding emerin. Defects of A-type lamins (or Lamin A and C), two other nuclear envelope proteins were identified shortly after as being responsible of the autosomal form of EDMD (EDMD2, OMIM#181350). The same gene, *LMNA* was then found mutated in a large spectrum of disorders, now called Laminopathies, affecting the skeletal and cardiac striated muscles, the peripheral nerves, the adipose tissue or leading to segmental premature ageing syndromes. These discoveries have shed light on the nuclear envelope, and mutations in genes encoding other nuclear envelope proteins were regularly reported in cascade during the last 15 years.

The French network on ‘EDMD & other nuclear envelope related diseases’ directed by Drs. Gisèle Bonne, Rabah Ben Yaou and France Leturcq (Paris), organizes annual meetings since it has been created in 2000. The Italian Network for Laminopathies, directed by Dr Giovanna Lattanzi (Bologna) and established in 2009, convene meetings twice a year. On January, 15-16, 2015, for the first time, the two networks held a joint meeting in Marseille at La Timone Adults’ Hospital: The 1st French-Italian meeting on laminopathies & other nuclear envelope-related diseases. This meeting was organized by Dr. Annachiara De Sandre-Giovannoli and Pr. Nicolas Lévy and the directors of the French and Italian networks. The meeting aimed to provide an update of recently acquired knowledge on: *i)* preclinical researches, *ii)* clinical researches, *iii)* patient registry and databases and *iv)* clinical trials in some of these rare diseases. The meeting also provided an understanding of the current state of the art on laminopathies and other nuclear envelope related diseases across France, Italy and the Iberian Peninsula and an opportunity to exchange ideas to improve patients’ healthcare organization in the future in a larger European/international context. The meeting has gathered 108 participants during two days. The first day was dedicated to communications among professionals involved in diagnosis, research and treatment of laminopathies and related diseases, and open to industrial partners, and the second day was dedicated to communications and view exchanges among professionals and patients’ families, aiming to inform them of the state of the art concerning their disease in terms of research and treatment. Among the invited speakers, Dr. Carlos Lopez-Otin, a leading scientist in the field of Progeria research, Dr. Raoul Hennekam, an expert clinician in the diagnosis and follow up of progeroid laminopathies and lipodystrophies, Dr. David Araujo-Vilar, the coordinator of the European consortium on lipodystrophies. These two days have been rich of view exchanges and informative for professionals and patients’ families, and have helped to further develop novel as well as already established fruitful collaborations.

It is planned not only to organize a second joint meeting between the French and Italian networks, but also to more widely open these meetings to other European colleagues, since already for this first edition, the Iberian community was largely represented. No doubt that this first edition will be the first one of a long series, since nuclear envelope proteins and their related diseases are extremely diverse and in continuous evolution.

